# Bacterial Spectrum and Antimicrobial Resistance Patterns of External Ocular Infections Among Patients Attending Dilla University General Hospital Ophthalmic Clinic, Southern Ethiopia

**DOI:** 10.1155/cjid/5593194

**Published:** 2025-06-27

**Authors:** Zerihun Solomon, Sara Anberbir, Gemechu Churiso, Asaye Mitiku, Alayu Bogale, Habtamu Gebrie, Samuel Tefera, Melkam Andargie, Mesfin Abebe

**Affiliations:** ^1^Department of Medical Laboratory Science, Dilla University, Dilla, Southern Ethiopia, Ethiopia; ^2^Department of Midwifery, Dilla University, Dilla, Southern Ethiopia, Ethiopia

**Keywords:** bacterial spectrum, dilla, ocular infections, ophthalmic clinic

## Abstract

Health professionals in ophthalmic clinics prescribe broad-spectrum topical antibiotics empirically, a major contributing factor to antimicrobial resistance. This practice is also observed in our study area. Thus, this study was done to identify the bacterial spectrum, determine antimicrobial resistance, and identify factors of external eye infections. A cross-sectional study was done from May to December 2023 with a systematic random sampling technique. The study participants' data were collected using a semistructured questionnaire. The specimen was taken aseptically and processed using standard microbiological methods. A total of 413 subjects were enrolled in this study. The overall prevalence of bacterial isolates was 52.8% (218/413) [95% CI: 48.0–57.6]. Gram-positive bacteria [70.6% (154/218)] predominate over gram-negative bacteria [29.4% (64/218)]. Considerable bacteria have shown a high percentage of resistance to penicillin and ampicillin. History of eye surface disease (AOR: 11.79, 95% CI: 2.79–49.69; *p* = 0.001) and previous usage of antibiotics (AOR: 3.47, 95% CI: 1.12–10.73; *p* = 0.031) have shown a significant association with bacteria isolated from the external part of the eye. The prevalence of bacteria isolated from the external eye was relatively high. Most bacteria have shown resistance to penicillin and ampicillin. Hence, antimicrobial susceptibility tests better monitor the empirical treatment of external eye infections.

## 1. Introduction

The eye, which is supposed to be impervious to most external agents, is possibly the most exposed structure among parts of the human body [1, 2]. In normal physiology, eye barriers such as the lids and tear film physically keep the eye, and the immune system, in combination with the microbiome, inhibits the growth of pathogenic microorganisms [2, 3]. Eye infections may occur when this physiology is disturbed because of underlying systemic diseases, trauma, surgery, eyeglass wearing, or several environmental factors [2, 3].

Although different eye components are prone to microorganisms, the most frequently infected parts are the external parts of the eye, encompassing the cornea, eyelid, and conjunctiva [3–5]. External eye infections (EEIs) may be clinically manifested as conjunctivitis, keratitis, dacryocystitis, endophthalmitis, and blepharitis; conjunctivitis being the most commonly encountered eye infection with perceptible economic and social impact [6, 7].

Many infectious agents may lead to EEIs, including bacteria, viruses, fungi, and parasites; however, bacterial infections contribute to up to 74% of eye infections worldwide [5, 8]. The commonest bacteria behind EEIs were Gram-positive bacteria such as *Staphylococcus aureus*, coagulase-negative staphylococci (CONS), *Streptococcus pneumoniae*, and Bacillus species, along with Gram-negative bacteria such as *Escherichia coli, Pseudomonas aeruginosa*, *Klebsiella pneumoniae*, Moraxella, and Haemophilus species [4, 6, 8, 9].

The morbidity of EEIs may range from self-limiting to blindness [9]. Globally, 2.2 billion people are visually impaired, among whom almost half could have been prevented, as stated by the World Health Organization report of 2023 [10]. In sub-Saharan Africa, approximately 26 million people live with vision impairment, with 5.9 million people being blind [11]. The epidemiological patterns of eye infections differ from one country to the other and even differ from one place to another within the same country [12]. In Ethiopia, for instance, the rate of blindness was stated to be 1.6%, of which 87.4% of them were because of infectious causes, which can be easily prevented [13].

Resistance to antimicrobial agents among bacteria is a priority public health concern, on the word of the report from the Centers for Disease Control and Prevention [14]. Studies reported that optometrists, ophthalmologists, and general practitioners working in ophthalmic clinics prescribe broad-spectrum topical antibiotics empirically based on findings from clinical examination without any laboratory results [15, 16]. This practice is also observed in our study area, as depicted by observational findings obtained during situational analysis of the current study. Empirical treatment of clinical cases with broad-spectrum antibiotics is a major contributing factor to antimicrobial resistance development [8]. Above all, the worsening scenario is that the study area is located only 400 km away from Gambo, Kenya, from where an enormous number of antibiotics are imported illegally through contraband. The community purchases those antibiotics just as routine merchandise without a prescription, which is one contributing factor to antimicrobial resistance [17, 18]. Antibiotic resistance in ophthalmology may not be life-threatening; however, it may result in treatment failures, which may result in devastating consequences like loss of sight [19, 20].

Although the bacterial spectrum causing EEIs is thoroughly studied, as evidenced by various literature [19, 21, 22], their distribution varies in different places, and even data from the same hospital collected at different times showed variation in the bacterial spectrum [12, 23]. This variation is attributed to regional and environmental factors, and seasonal changes [24, 25]. Similarly, antimicrobial resistance may differ significantly with time and geographical variation, mainly because of intense antibiotic utilization, which enhances the resistant bacteria [26].

Therefore, periodic surveillance of the bacterial spectrum and antimicrobial resistance patterns of those bacteria in different places is crucial to keeping health professionals and other stakeholders up to date with proper antibiotic therapy for appropriate medical intervention of EEIs [27]. Moreover, to our knowledge, no study has been conducted investigating the bacterial spectrum, antimicrobial resistance patterns, and associated factors of EEI in this specific catchment area. Thus, this study aimed to fill this critical gap by identifying the bacterial spectrum of the external part of the eye, determining antimicrobial resistance patterns, and identifying factors of EEIs among clients attending Dilla University General Hospital's ophthalmic unit.

## 2. Materials and Methods

### 2.1. Study Design, Area, and Period

A cross-sectional study was done from May to December 2023 at Dilla University General Hospital, southern Ethiopia, which is located in Dilla town. The town is the chief town of the Gedeo zone, which is found in the newly formed southern Ethiopia Region. It is situated 355 km south of Addis Ababa and 80 km away from Hawassa. The estimated total population of the town is 954, 120 according to the 2007 Ethiopian Central statistical agency report [28]. There is one public hospital (Dilla University General Hospital) in Dilla town that offers health services for more than two million people of Gedeo and Amaro kele Zone from the south Ethiopia region, and neighboring regions like Sidama and Oromia. The hospital delivers preventive, curative, and rehabilitative care in different departments including the ophthalmic clinic to the population in the catchment area. The ophthalmic clinic serves nearly 100 patients daily with various ophthalmic cases.

### 2.2. Populations

All new and returning clients of any age who visited Dilla University General Hospital's ophthalmic unit were considered as the source population. Clients diagnosed by an ophthalmologist for EEIs clinically, based on standard clinical criteria in the period of the study, were included as the study population. Clients who had taken antibiotics in the last 2 weeks and those who had had ocular surgery within 1 week before recruitment of the study participants were excluded.

### 2.3. Study Variables

The bacterial spectrum of EEIs was the outcome variable, whereas age, residence, educational level, cosmetic application practices, cigarette smoking habit, face washing, comorbid condition, eye surface diseases, duration of current illness, previous use of antibiotics, history of eye trauma, use of digital screen (computer, mobile phone, television, etc.) and history of hospitalization were independent variables.

### 2.4. Sample Size and Sampling Technique

#### 2.4.1. Sample Size

The single population proportion formula was used to calculate the sample size for the study. The assumptions made were 48.8% prevalence from Hawassa, Ethiopia [29], 95% certainty, and the margin of sampling error tolerated was 5%. After adding a 10% nonresponse rate and computing the calculation, the sample size of the study was 422.

#### 2.4.2. Sampling Technique

The sampling technique of the study was a systematic random sampling technique. It was used to choose the study subjects by calculating the *k*th value, where *N* = 1350 (based on the average data obtained from Dilla University General Hospital ophthalmic unit, in the year 2022, within seven consecutive months (May–December).

The *K*th values are determined accordingly.

(1) $$\begin{array}{c} K^{\text{th}}\text{ value}=\frac{N}{n}=\frac{1350}{422}∼3. \end{array}$$

Based on this, every third study participant was selected. The first study subject was chosen with a lottery method from 1 to 3 patients and became the third patient. Thereafter, every third patient who came to the facility was included.

### 2.5. Data Collection and Laboratory Investigation

#### 2.5.1. Sociodemographic and Clinical Data Collection

After the coming of each study subject, he/she was informed to give written assent/consent provided that the study objectives were explained. Data on clinical and sociodemographic characteristics of the study subjects were asked and collected by trained optometrists through face-to-face interviews using semistructured questionnaires and retrieving patients' medical records.

#### 2.5.2. Specimen Collection, Transportation, and Handling

The clinical diagnosis of EOI was made by an ophthalmologist using a slit lamp biomicroscope [30]. The client was invited to observe the roof, the specimen was obtained using a swab moistened in sterile saline by pulling down the lower eyelid and rubbing from the medial to the lateral side of the lower conjunctival sac [13, 31]. In the case of dacryocystitis lacrimal sac was the area of choice from where the pus was taken, while discharge was collected using a dry cotton swab (sterile) from the margin of the eyelid in the case of blepharitis [9, 32]. Then, the cotton swab was submerged in 3 mL of brain heart infusion (BHI) broth and taken for examination to the Dilla University General Hospital microbiology laboratory [33].

#### 2.5.3. Bacterial Isolation and Identification

The specimens from BHI broth were inoculated on blood agar, mannitol salt agar, MacConkey, and chocolate agar and incubated at 35°C–37°C for 24 h. Blood agar plates (BAP) and chocolate agar plates (CAP) were incubated in an anaerobic candle jar to enhance the growth of fastidious bacteria, maintaining 5%–10% CO_2_. Those culture media without bacterial growth were further incubated for 48 h. After taking pure bacterial colonies, further identification of the isolates was conducted by using Gram staining and biochemical tests. Catalase, coagulase, bacitracin, and optochin tests were used for isolation and identification of Gram-positive cocci, whereas biochemical tests, like killer iron agar (KIA), lysine decarboxylase agar (LDC), Simmon's citrate test, urease test, motility, hydrogen sulfide, and indole tests, were used for identification of Gram-negative bacterial isolates [34, 35].

#### 2.5.4. Antimicrobial Susceptibility Testing

Kirby–Bauer disc diffusion technique was used for antimicrobial susceptibility testing based on Clinical Laboratory Standard Institute (CLSI) 2021 guideline on Muller–Hilton agar (MHA) for nonfastidious bacteria; or for fastidious bacteria like *Streptococcus pneumoniae,* MHA added with 5% sheep blood (Oxoid Ltd) was used [36]. The bacterial suspension was prepared by taking 3–5 pure colonies with a sterile wire loop, mixing in 3 mL of physiological normal saline (0.85% NaCl) gently to adjust the suspension to 0.5 McFarland's standard. The cotton swab (sterile) was immersed in the suspension of the bacteria, and the excess fluid was removed by pressing it against the surface of the container. The swab was then consistently rubbed to the whole surface of MHA. The plates were put at room temperature for 3–5 min to dry up.

Gram-positive isolates were tested against the following antibiotics each from Oxoid Ltd. (United Kingdom): penicillin 10units, ampicillin 10 μg, vancomycin 30 μg, ceftriaxone 30 μg, chloramphenicol 30 μg, erythromycin 15 μg, tetracycline 30 μg, clindamycin 2 μg, cefoxitin 30 μg, amoxicillin clavulanate 20 μg, ciprofloxacin 5 μg, gentamicin 10 μg, trimethoprim–sulfamethoxazole 1.25/23.75 μg, and meropenem 10 μg. *S. aureus* and CONS were assessed against cefoxitin 30 μg to determine the methicillin resistance pattern of those bacteria. Then again, Gram-negative bacteria were tested against ciprofloxacin 30 μg, gentamicin 10 μg, tetracycline 30 μg, trimethoprim/sulfamethoxazole 1.25/23.75 μg, meropenem 10 μg, amikacin 30 μg, ampicillin 10 μg, amoxicillin clavulanic acid 20 μg, ceftazidime 30 μg, and ceftriaxone 30 μg (Oxoid Ltd). The discs were put on the MHA surface using sterilized forceps provided that each was 15 mm apart from the other to avoid overlapping of zone of inhibition. The plates were permitted to stand for 15 min to dissolve the antibiotics and put in an incubator for 18–24 h at 37°C. Results were reported as sensitive, intermediate, and resistant based on CLSI guidelines [37]. The antimicrobial discs were chosen by CLSI recommendation and commonly prescribed antibiotics in the Dilla University General Hospital ophthalmic unit.

#### 2.5.5. Data Quality Control

To keep consistency throughout the data collection, the questionnaire, which was organized in English, was translated into Amharic and *Gedeuffa* languages and retranslated back to English. A pretest was conducted on 5% (*n* = 21) of the sample to safeguard the quality of data at Yirgalem General Hospital, Sidama, Ethiopia. Data collectors and supervisors have taken 2 days of training to minimize interpersonal variation during data collection. All data were patterned for the entirety, and the necessary reaction was sent back to the data collectors immediately. All procedures in each stage adhered to standard operating procedures (SOPs). A sterility check was performed on 5% of each batch of media preparation to avoid contamination. All reagents were made ready consistent with the manufacturer's instructions and checked for their expiry date. Moreover, *Streptococcus pyogenes* (ATCC 19615) and *Staphylococcus aureus* (ATCC 25923) strains for Gram-positive bacteria, and *Pseudomonas aeruginosa* (ATCC 27853) and *Escherichia coli* (ATCC 25922) for Gram-negative bacteria were used to check the quality of media prepared and the antibiotic discs used in the study. The reference strains were obtained from the Ethiopian Public Health Institute (EPHI).

### 2.6. Statistical Analysis

Data were edited, coded, and entered by Epi-Data Version 4.6.0.2 and imported into Statistical Package for Social Sciences (SPSS) Version 25 for analysis. Patients' demographics and other characteristics were narrated using descriptive statistics. The presence of association between outcome and independent variables was determined by using both bivariate and multivariable logistic regression analysis. Initially, the data were analyzed through bivariate analysis; then, those variables at a cutoff point *p*-value ≤ 0.25 were candidates for multivariable analysis; 95% confidence interval (CI) and adjusted odds ratio (AOR) were used to assess and measure the strength of association between outcome and independent variables. *p*-value ≤ 0.05 in the multivariable analysis was taken as statistically significant. Lastly, the findings were shown by texts, graphs, and tables.

## 3. Result

### 3.1. Sociodemographic Characteristics of the Study Subjects

A total of 413 study subjects were included in this study, with a 97.87% (413/422) response rate. The mean age of the enrolled study subjects was 36.15 with a standard deviation of ± 16.03 years. More than half of the study participants were females (222/413; 53.8%). One-fourth of the study participants (108/413; 25.90%) were unable to read and write. More than half (232/413; 56.20%) of the participants were rural residents, and nearly one-third (117/413; 28.30%) of them were farmers (Table 1).

### 3.2. Clinical Characteristics of the Study Subjects

The majority of the study subjects did not wear contact lenses (379/413; 91.80%). Nearly half (203/413; 49.20%) of the study participants had a history of eye surface disease, and a history of hospital admission (209/413; 50.60%). On the other hand, about one-third (130/413; 30.50%) of the study subjects had a history of eye trauma. More than half (220/413; 53.3%) of the study participants used antibiotics previously, while the majority of them (323/413; 78.20%) did not have comorbid conditions like diabetes mellitus, hypertension, renal diseases, and/or heart disease.

### 3.3. Prevalence of Bacterial Isolates of External Eye Infections

The overall prevalence of bacteria from patients clinically diagnosed with EEIs in the current study was 52.8% (218/413) [95% CI: 48.0–57.6]. Gram-positive bacteria predominate over Gram-negative bacterial isolates with respective percentages of 70.6% (154/218) and 29.4% (64/218). Besides, various Gram-positive bacterial species were isolated with different percentages, the highest being *Staphylococcus aureus* 47.4% (73/154), followed by CONS 31.2 (48/154), *Streptococcus pneumoniae* 11.0% (17/154), *Streptococcus pyogenes* 5.8 (9/154), and *Enterococcus* species 4.5% (7/154).

Likewise, different species of Gram-negative bacterial isolates were observed, with the highest percentage seen for *Escherichia coli* at 25% (16/64) followed by *Pseudomonas aeruginosa* at 21.9% (14/64), *Klebsiella pneumoniae* at 18.8% (12/64), *Proteus* species at 12.5% (8/64), and *Haemophilus influenzae* and *Moraxella catarrhalis*, each with a percentage of 10.9% (7/64).

**Figure 1:**
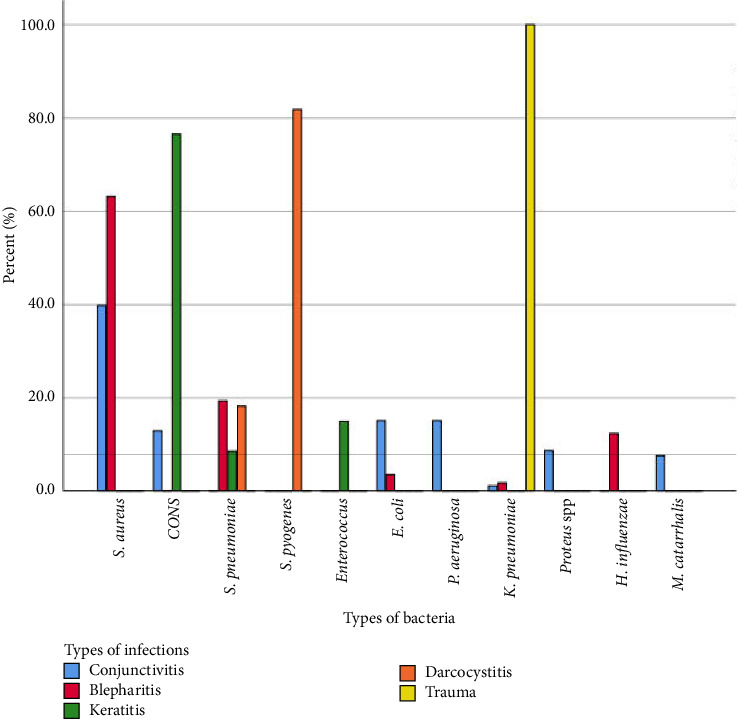
Types and percentage of bacteria isolated from various types of external eye infections among patients who attended the DUGH ophthalmic clinic, Southern Ethiopia.

In terms of types of EEIs diagnosed clinically, the percentage of bacterial isolate was 42.7% (93/218) for conjunctivitis, 26.1% (57/218) for blepharitis, 21.6% for keratitis (47/218), 5.0% (11/218) for dacryocystitis, and 4.6% (10/218) for trauma. Various bacterial isolates have contributed to each type of EEI. For instance, conjunctivitis was caused by a variety of Gram-positive and Gram-negative bacteria, the highest being *S. aureus* 39.8% (37/93), then *E. coli* and *P. aeruginosa* each contributing with a percentage of 15.1% (14/93) followed by CONS 12.9% (12/93), *Proteus* species 8.6% (8/93), *M. catarrhalis* 7.5% (7/93), and *K. pneumoniae* 1.1% (1/93). Similarly, blepharitis was also caused by both Gram-positive and Gram-negative bacteria with the highest percentage reported for *S. aureus* 63.2% (36/57), followed by *S. pneumoniae* 19.3% (11/57), *H. influenzae* 12.3% (7/57), *E. coli* 3.5% (2/57), and *K. pneumoniae* 1.8% (1/57). Nevertheless, keratitis and dacryocystitis were caused by only Gram-positive bacteria and the etiologic agents were CONS 76.6% (36/47), *Enterococcus* species 14.9% (7/47), and *S. pneumoniae* 8.5% (4/47) for keratitis, while for that of dacryocystitis it were *S. pyogenes* 81.8% (9/11) and *S. pneumoniae* 11.8% (2/11). On the other hand, a single bacterial species (*K. pneumoniae*) was isolated from patients affected by trauma (Figure 1).

### 3.4. Antimicrobial Resistance Patterns of Gram-Positive Bacteria

The antibiotic resistance patterns of Gram-positive bacteria were assessed against fourteen commonly prescribed antibiotics. Accordingly, each isolate has shown a different resistance pattern for the tested antibiotics. For instance, *S. aureus* and CONS have shown the highest resistance at 98.6% (72/73) and 97.9% (47/48) to penicillin, respectively, followed by ampicillin at 97.3% (71/73) for *S. aureus* and 93.8% (45/48) for CONS. However, *S. aureus* has shown the lowest resistance to vancomycin, 98.6% (72/73), while none of the CONS isolates have shown resistance to vancomycin and ciprofloxacin (100% sensitive). The antimicrobial susceptibility test made against cefoxitin has indicated that *methicillin-resistant Staphylococcus* (MERSA) was observed among 30.1% (22/73) of *S. aureus* isolates and 10.4% (5/48) of CONS isolates. Likewise, *Enterococcus* species have shown the highest resistance (100%) to both penicillin and ampicillin, but the lowest resistance was observed to vancomycin, 14.3% (1/7). Put the matter another way, *vancomycin-resistant Enterococcus* (VRE) was observed in 14.3% (1/17) of the *Enterococcus* isolates. On the other hand, *S. pneumoniae* has shown little resistance, whereas *S. pyogenes* has demonstrated almost no resistance to the tested antibiotics (Table 2). A few of the *S. pneumoniae* isolates were observed to be intermediate for some of the tested antibiotics.

### 3.5. Antimicrobial Resistance Patterns of Gram-Negative Bacterial Isolates

Gram-negative bacteria were also tested against various commonly prescribed antibiotics (eleven antibiotics) and showed different resistance patterns. *E. coli* isolates have shown the highest resistance to ampicillin at 87.5% (14/16), but the lowest resistance (100% sensitive) to gentamicin and meropenem antimicrobials. Unfortunately, resistance to most of the tested antibiotics was observed among isolates of *P. aeruginosa,* the highest being trimethoprim–sulfamethoxazole at 78.6% (11/14). Nevertheless, these isolates have shown the lowest resistance to meropenem at 7.1% (1/14) followed by ciprofloxacin at 21.4% (3/14). *K. pneumoniae* isolates have also shown the highest resistance to ampicillin, 91.7% (11/12), but the lowest resistance to meropenem (100% sensitive), and ciprofloxacin and gentamicin, each showing 8.3% (1/12) resistance. A few isolates of Gram-negative bacteria have shown intermediate resistance patterns to some of the tested antibiotics (Table 3).

### 3.6. Multidrug Resistance (MDR) Patterns of Isolated Bacteria

**Figure 2:**
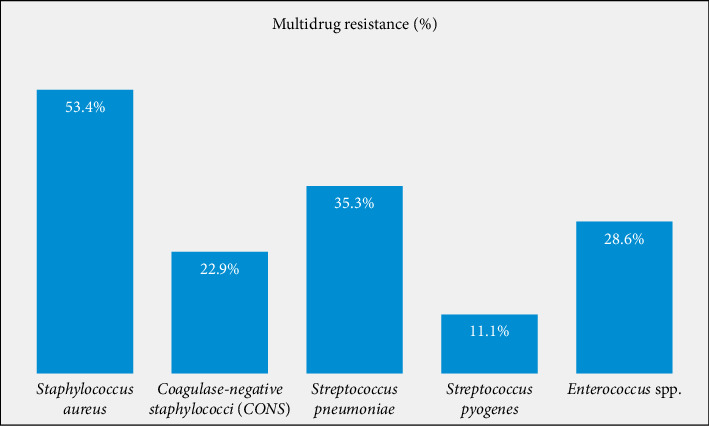
Multidrug resistance patterns of Gram-positive bacterial isolates among patients attending DUGH ophthalmic clinic, Southern Ethiopia, 2023.

**Figure 3:**
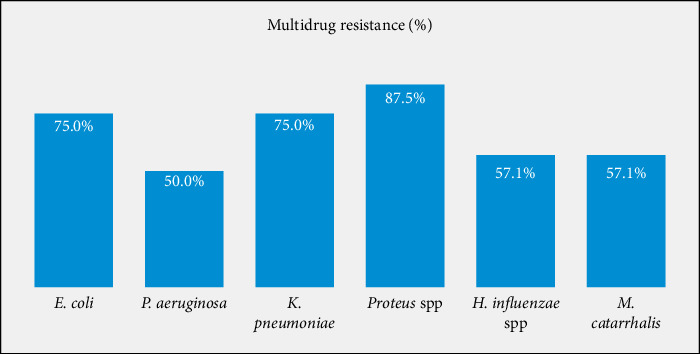
Multidrug resistance patterns of Gram-negative bacterial isolates among patients attending DUGH ophthalmic clinic, Southern Ethiopia, 2023.

The overall percentage of multidrug-resistant bacterial isolates was 46.8% (102/218). About 38.3% (59/154) of the Gram-positive bacterial isolates showed an MDR pattern, *S. aureus* showing the highest percentage of resistance [53.4% (39/73)] (Figure 2). Among Gram-negative bacterial isolates, 67.2% (43/64) showed an MDR pattern, *E. coli* [75% (12/16)] and *K. pneumoniae* [75% (9/12)] showing the highest percentage (Figure 3).

### 3.7. Factors Associated With Bacterial Isolates of External Eye Infections

Various sociodemographic, behavioral, and clinical variables of study subjects were identified and assessed against bacterial isolates of EEIs for possible association using a logistic regression model. Accordingly, in bivariate analysis, age (25–64 age group *p* = 0.205), educational level (college and above *p* = 0.190), occupation (farmer *p* = 0.188 and merchant *p* = 0.095), history of the eye surface disease (*p* = 0.177), previous usage of antibiotics (*p* = 0.006), duration of illness (2–4 weeks *p* = 0.020), and comorbid conditions (*p* = 0.044) are considered as candidates for multivariable logistic regression provided that the cutoff value was *p* ≤ 0.25 (Table 4). However, after multivariable analysis, only history of eye surface disease (AOR: 11.79, 95% CI: 2.79–49.69; *p* = 0.001) and previous usage of antibiotics (AOR: 3.47, 95% CI: 1.12–10.73; *p* = 0.031) have shown significant association with bacterial isolates of EEIs (Table 5).

## 4. Discussion

The overall prevalence of bacteria in the current study was 52.8% (218/413) [95% CI: 48.0–57.6]. The result was lower than studies conducted in Greece (63.4%) [16], China (82.7%) [23], Nigeria (88.6%) [37], Sudan (63.7%) [38], Uganda (69%) [39], Ghana (95%) [40], and Felege Hiwot (57.8%) [4], and Gondar (58.3%) [1], northwest Ethiopia; Jijiga (62.2%) [9], East Ethiopia; and Hawassa (48.8%) [29], south Ethiopia. However, the result is higher than previous studies done in Naples, Italy [41], Bangalore, India (34.5%) [3], and Jimma, southwest Ethiopia (46.1%) [42]. The discrepancy in the results might be attributed to different factors like variation in study participants, geographical differences, and adherence differences to infection prevention protocols, which might, in turn, be attributed to variations in population for access to health education, community awareness, and countries' developmental level.

The current study also revealed that Gram-positive bacteria 70.6% (154/218) predominate Gram-negative bacteria 29.4% (64/218), and the result is consistent with other studies previously conducted in Italy [6], Bangalore, India [3], Iran [22], Riyadh Saud Arabia [43], Nigeria [37], and Ethiopia [4, 13, 21]. Nevertheless, it disagrees with findings from Sudan [38], where Gram-negative bacteria predominate over Gram-positive bacteria. The discrepancy might be due to differences in a microbiology laboratory setup, availability of resources, types of culture media used for bacterial isolation, and sample size differences (the current study has used a larger sample size).

Additionally, the study indicated that among Gram-positive bacterial isolates, the highest percentage was *Staphylococcus aureus*, 47.4% (73/154). This finding was in agreement with previous studies done in the United States [2], Greece, Nigeria [16], and Ethiopia [7, 13]. But it was inconsistent with other studies done in Italy [6], China [23], Riyadh, Saudi Arabia [43], and Uganda [39], where the predominant isolates were CONS; and Iran [22], where *P. aeruginosa* isolates predominate. The difference could arise from differences in study participants, variations in the type of normal flora harbored by the patients, which may in turn become a source of infection [44], and variations in personal hygiene protection habits.

Likewise, different species of Gram-negative bacterial isolates were observed, with the highest percentage seen for *Escherichia coli* at 25% (16/64). This result was similar to other studies done previously in Italy [6], and Shashamane, Ethiopia [45]. Nonetheless, it was contrary to other findings from Nigeria [37] and Sudan [38] where dominance was observed by *Haemophilus influenzae;* Mexico [46], Bangalore, India [3], Iran [22], and China [23] where Pseudomonas species were reported predominantly; and Felege Hiwot Hospital, northwest Ethiopia, where *K. pneumoniae* was seen with the highest percentage [4]. The justification might be that various infectious agents have variable epidemiological distributions, although most of them are ubiquitous.

Regarding types of EEIs diagnosed clinically, the majority of the bacterial isolates were detected from conjunctivitis [42.7% (93/218)] followed by blepharitis [26.1% (57/218)], keratitis [21.6% (47/218)], dacryocystitis [5.0% (11/218)], and trauma [4.6% (10/218)]. This finding was in agreement with other studies done in northwest Ethiopia. The distributions suggest that bacterial isolates of external ocular infections are mostly isolated from conjunctivitis.

Gram-positive bacteria have shown the highest resistance to penicillin and ampicillin but the lowest resistance to vancomycin and ciprofloxacin, which is consistent with other studies conducted in Greece, Italy [6], Sudan [38], Addis Ababa [7], and Felege Hiwot, northwest Ethiopia [4]. The resistance to penicillins could be justified because most Gram-positive bacteria produce β-lactamase enzymes that can degrade the β-lactam ring of penicillin antibiotics or inhibit their penicillin-binding proteins (PBP) either through alteration of the inherent PBP genes or getting external DNA [47].

MERSA was observed among 30.1% (22/73) of *S. aureus* isolates and 10.4% (5/48) of CONS isolates. The result was in agreement with a study conducted in Uganda, 31.9% (29/91) for MERSA [39]. However, the finding was slightly larger than those conducted in Naples, Italy [48], Gondar, northwest Ethiopia (24%) [1]; and Jimma, southwest Ethiopia (13.8%) [42]. The increase in the result might be due to the larger sample size used in the current study (a large proportion of *S. aureus* and CONS were isolated). On the contrary, it was lower than the study done in Jinka, southern Ethiopia, which was 45.6% and 36.8% for MERSA and CONS, respectively [5]. The possible reasons might be differences in antibiotic usage practice, personal hygiene protection, sanitary conditions of the living environment, and economic status of the community.

Besides, most Gram-negative bacteria have shown the highest resistance to ampicillin [*K. pneumoniae* 91.7% (11/12), (*E. coli* 87.5% (14/16), *M. catarrhalis* 71.4% (5/7), *Proteus* spp. 37.5% (3/8), and *H. influenzae* 28.6% (2/7)], but lowest resistance to meropenem (0%–10%), gentamicin (0%–10%), and ciprofloxacin (10%–20%). The finding was supported by other findings from Greece [16] and Addis Ababa, Ethiopia [7]. The resistance of Gram-negative bacteria to ampicillin is due to the ability of the bacteria to produce a ß-lactamase enzyme that cleaves the ß-lactam ring of penicillin antibiotics although the way of getting this enzyme is different for the various types of Gram-negative bacteria; *K. pneumoniae* for instance produce SHV-1 penicillinase in their chromosome [49], while *E. coli* and *H. influenzae* produce TEM-1 β-lactamase, which is a form of class A enzyme encoded by a plasmid [50, 51]. Besides, *Proteus* spp. acquire chromosomal β-lactamase expression and β-lactamase production mediated by plasmid [52], and *M. catarrhalis* produces BRO-1 and BRO-2 β-lactamase encoded by two respective genes named bro-1 and bro-2 [53].

The overall percentage of multidrug-resistant bacterial isolates in this study was 46.8% (102/218). This finding was lower than reports from Gondar (64.6%), Addis Ababa (66.4%), and Debre Markos, Ethiopia (59.2%) [1, 8, 54]. But, it is higher than findings from Western Greece (4.2%) and Bahir Dar (45.2%), northwest Ethiopia [4]. The observed multidrug-resistant trend of the bacterial isolates to different antibiotics could be attributed to the use of broad-spectrum antibiotics, not regularly checking for antimicrobial resistance patterns before prescription, self-medicating, and misusing drugs [4, 8].

This study also indicated that patients with a previous history of eye surface disease were eleven times more likely to harbor bacterial isolates of EEIs compared with patients who did not have a history of eye surface disease (AOR: 11.79, 95% CI: 2.79–49.69; *p* = 0.001) which was in agreement with former studies done elsewhere [4, 55, 56]. The reason might be because previously compromised external eye components, especially the cornea, have an association with bacterial eye infections [55].

Likewise, patients having a history of antibiotic usage were three times more likely to harbor bacterial isolates of EEIs when compared with those who did not have a history of antibiotic usage (AOR: 3.47, 95% CI: 1.12–10.73; *p* = 0.031). This result was also aided by other studies done in Gondar, northwest Ethiopia [1], and Jinka, southern Ethiopia [5]. The justification might be because previous exposure to antimicrobials disrupts the normal flora that competes for nutrients and space, thereby creating an appropriate milieu for the overgrowth of pathogenic microorganisms [57, 58]. Moreover, the normal flora is assumed to play a great role in enhancing the immune system of the host and hence the response made against pathogens [57, 59].

## 5. Conclusion

The prevalence of bacteria in external ocular infections was relatively high in the current study setting. Gram-positive bacteria predominantly cause external ocular infections. Most bacteria (both Gram-positive and Gram-negative) were resistant to penicillin and ampicillin. However, Gram-positive bacteria showed the lowest percentage of resistance to vancomycin, ciprofloxacin, and gentamycin, while meropenem, gentamycin, and ciprofloxacin were antibiotics with the lowest resistance against Gram-negative bacteria. A considerable number of *S. aureus* and CONS isolates were methicillin-resistant. Other tested antibiotics have shown variable percentages of resistance patterns against each isolate. Having a history of eye surface disease and a history of antibiotic usage were factors significantly associated with bacterial isolates of EEIs. Therefore, empirical treatment of external ocular infections is better reduced and substituted by antimicrobial susceptibility tests to lessen the resistance of bacterial isolates to antibiotics. Besides, those individuals having a history of eye surface diseases should follow up in nearby health institutions to alleviate the chance of getting infected with EEIs.

## Figures and Tables

**Table 1:** Sociodemographic and clinical characteristics of patients attending DUGH ophthalmic clinic from May to December 2023.

Variables	Categories	Frequency (*n* = 413)	Percentage (%)
Age	< 5	30	7.3
	5–14	64	15.5
	15–24	23	5.6
	25–34	95	23.0
	35–44	96	23.2
	45–54	62	15.0
	55–64	22	5.3
	≥ 65	21	5.1

Sex	Female	222	53.8
	Male	191	46.2

Residence	Urban	181	43.8
	Rural	232	56.2

Educational level	Unable to read and write	107	25.9
	Primary	108	26.2
	Secondary	125	30.3
	College and above	73	17.7

Occupation	Civil servant	90	21.8
	Farmer	117	28.3
	Merchant	67	16.2
	Housewife	48	11.6
	Student	77	18.6
	Other	14	3.4

Contact lens wearing	Yes	34	8.20
	No	379	91.80

Frequency face washing	Frequent	149	36.10
	Once a day	137	33.20
	Less frequent	127	30.70

History of eye surface disease	Yes	203	49.20
	No	210	50.80

Hospital admission ever?	Yes	209	50.60
	No	204	49.40

History of eye trauma	Yes	130	31.50
	No	283	68.50

Previous use of antibiotics	Yes	220	53.30
	No	193	46.70

Duration of illness (*n* = 220)	< 1 week	44	20.00
	2–4 week	78	35.50
	> 4 week	98	45.50

Comorbid condition	Yes	90	21.80
	No	323	78.20

Use of a digital screen	Yes	67	16.20
	No	346	83.80

Eye cosmetics usage	Yes	36	8.70
	No	377	91.30

**Table 2:** Antimicrobial resistance patterns of Gram-positive bacterial isolates observed among patients attending DUGH ophthalmic clinic, Southern Ethiopia, 2023.

Antibiotics	ASP (%/*n*)	Types of Gram-positive bacteria				
		*S. aureus* (*n* = 73)	*CONS* (*n* = 48)	*S. pneumoniae* (*n* = 17)	*S. pyogenes* (*n* = 9)	*Enterococcus* spp (*n* = 7)
PEN	S	1.4 (1)	2.1 (1)	11.8 (2)	88.9 (8)	0
	I	0	0	0	0	0
	R	98.6 (72)	97.9 (47)	88.2 (15)	11.2 (1)	100 (7)

AMP	S	—	—	—	88.9 (8)	0
	I	—	—	—	0	0
	R	—	—	—	11.1 (1)	100 (7)

CTR	S	—	—	—	100 (9)	71.4 (5)
	I	—	—	—	0	0
	R	—	—	—	0	28.6 (2)

VAN	S	98.6 (72)	100 (48)	100 (17)	100 (9)	85.7 (6)
	I	0	0	0	0	0
	R	1.4 (1)	0	0	0	14.3 (1)

ERY	S	50.7 (37)	75 (36)	76.5 (13)	100 (9)	—
	I	0	0	5.9 (1)	0	—
	R	36 (49.3)	25 (12)	17.6 (3)	0	—

TET	S	34 (46.6)	77.1 (37)	52.9 (9)	77.8 (7)	—
	I	0	0	11.8 (2)	0	—
	R	39 (53.4)	22.9 (11)	35.3 (6)	22.2 (2)	—

CHL	S	56.2 (41)	75.0 (36)	23.5 (4)	88.9 (8)	57.1 (4)
	I	0	0	0	0	0
	R	43.8 (32)	25.0 (12)	76.5 (13)	11.1 (1)	42.9 (3)

CND	S	69.9 (51)	89.6 (43)	82.4 (14)	100 (9)	—
	I	0	0	17.6 (3)	0	—
	R	30.1 (22)	10.4 (5)	0	0	—

CFT	S	69.9 (51)	85.4 (41)	—	—	—
	I	0	0	—	—	—
	R	30.1 (22)	14.6 (7)	—	—	—

CN	S	61.6 (45)	93.7 (45)	—	—	—
	I	0	0	—	—	—
	R	38.4 (28)	6.3 (3)	—	—	—

AUG	S	—	—	100 (17)	—	—
	I	—	—	0	—	—
	R	—	—	0	—	—

STX	S	15.1 (11)	77.1 (37)	17.6 (3)	55.6 (5)	—
	I	0	0	5.9 (1)	0	—
	R	84.9 (62)	22.9 (11)	76.5 (13)	44.4 (4)	—

CPR	S	89.0 (68)	100 (48)	—	—	71.4 (5)
	I	0	0	—	—	0
	R	11.0 (5)	0	—	—	28.6 (2)

MER	S	—	—	100 (17)	100 (9)	—
	I	—	—	0	0	—
	R	—	—	0	0	—

*Note:* PEN = penicillin, AMP = ampicillin, CTR = ceftriaxone, VAN = vancomycin, ERY = erythromycin, TET = tetracycline, CHL = chloramphenicol, CND = clindamycin, CFT = cefoxitin, CN = gentamicin, AUG = augmentin, STX = trimethoprim/sulfamethoxazole, CPR = ciprofloxacin, MER = meropenem, CONS = coagulase-negative staphylococci.

Abbreviations: ASP = antimicrobial susceptibility patterns, I = intermediate, S = sensitive, R = resistant.

**Table 3:** Antimicrobial resistance patterns of Gram-negative bacterial isolates observed among patients attending DUGH ophthalmic clinic, Southern Ethiopia, 2023.

Anti-biotics	ASP [*n* (%)]	Types of Gram-negative bacteria					
		*E. coli* (*n* = 16)	*P. aeruginosa* (*n* = 14)	*K. pneumoniae* (*n* = 12)	*Proteus* spp (*n* = 8)	*H. influenzae* spp (*n* = 7)	*M. catarrhalis (n =* 7)
AMP	S	12.5 (2)	—	8.3 (1)	62.5 (5)	71.4 (5)	2 (28.6)
	I	0	—	0	0	0	0
	R	87.5 (14)	—	91.7 (11)	37.5 (3)	28.6 (2)	71.4 (5)

CTR	S	81.2 (13)	—	75.0 (9)	25.5 (2)	71.4 (5)	71.4 (5)
	I	0	—	0	0	0	0
	R	18.8 (3)	—	25.0 (3)	75.0 (6)	28.6 (2)	28.6 (2)

TET	S	75.0 (12)	—	33.3 (4)	12.5 (1)	—	85.7 (6)
	I	0	—	0	0	—	0
	R	25.0 (4)	—	66.7 (8)	87.5 (7)	—	14.3 (1)

CHL	S	50.0 (8)	—	16.7 (2)	25.0 (2)	85.7 (6)	14.3 (1)
	I	18.8 (3)	—	0	0	0	0
	R	31.2 (5)	—	83.3 (10)	75.0 (6)	14.3 (1)	85.7 (6)

CN	S	100 (16)	64.3 (9)	91.7 (11)	62.5 (5)	100 (7)	100 (7)
	I	0	0	0	0	0	0
	R	0	35.7 (5)	8.3 (1)	37.5 (3)	0	0

AUG	S	81.2 (13)	—	83.3 (10)	75.0 (6)	85.7 (6)	100 (7)
	I	0	—	0	0	0	0
	R	18.8 (3)	—	16.7 (2)	25.0 (2)	14.3 (1)	0

STX	S	25.0 (4)	—	58.3 (7)	62.5 (5)	28.6 (2)	71.4 (5)
	I	18.8 (3)	—	0	0	0	0
	R	56.2 (9)	—	41.7 (5)	37.5 (3)	71.4 (5)	28.6 (2)

CPR	S	93.7 (15)	78.6 (11)	91.7 (11)	87.5 (7)	85.7 (6)	85.7 (6)
	I	0	0	0	0	0	0
	R	6.3 (1)	21.4 (3)	8.3 (1)	12.5 (1)	14.3 (1)	14.3 (1)

MER	S	100 (16)	92.9 (13)	100 (12)	100 (8)	100 (7)	100 (7)
	I	0	0	0	0	0	0
	R	0	7.1 (1)	0	0	0	0

CZD	S	62.5 (10)	50.0 (7)	66.7 (8)	50.0 (4)	57.1 (4)	57.1 (4)
	I	0	0	0	0	0	0
	R	37.5 (6)	50.0 (7)	33.3 (4)	50.0 (4)	42.9 (3)	42.9 (3)

AMK	S	81.2 (13)	57.1 (8)	66.7 (8)	50.0 (4)	71.4 (5)	71.4 (5)
	I	6.3 (1)	7.1 (1)	8.3 (1)	25.0 (2)	0	0
	R	12.5 (2)	35.7 (5)	25.0 (3)	25.0 (2)	28.6 (2)	28.6 (2)

*Note:* AMP = ampicillin, CTR = ceftriaxone, TET = tetracycline, CHL = chloramphenicol, CN = gentamicin, AUG = augmentin, STX = trimethoprim/sulfamethoxazole, CPR = ciprofloxacin, MER = meropenem, AMK = amikacin, CZD = ceftazidime.

Abbreviations: ASP = antimicrobial susceptibility patterns, I = intermediate, S = sensitive, R = resistant.

**Table 4:** Bivariate logistic regressions of Sociodemographic, clinical, and behavioral factors assessed against culture-confirmed external eye infections at DUGH ophthalmic clinic, Southern Ethiopia, 2023.

Variables	Categories	Bacterial isolates *N* (%)		COR (95% CI)	*p*-value
		Yes	No		
Age	≤ 14	49 (22.5)	60 (30.8)	1	
	15–24	20 (9.2)	12 (6.2)	1.1O8 (0.536–0.2290)	0.782
	25–64	130 (59.6)	102 (52.3)	0.543 (0.211–1.400)	0.205^∗^
	≥ 65	19 (8.7)	21 (10.8)	0.710 (0.362–1.391)	0.318

Sex	Female	119 (54.6)	103 (52.8)	1.074 (0.729–1.582)	0.719
	Male	99 (45.4)	92 (47.2)	1	

Residence	Urban	100 (45.9)	81 (41.5)	1.193 (0.808–1.762)	0.376
	Rural	118 (54.1)	114 (58.5)	1	

Educational level	Illiterate	48 (22.1)	59 (30.3)	1	
	Primary	55 (25.2)	53 (27.2)	1.335 (0.735–2.424)	0.343
	Secondary	77 (35.3)	48 (24.6)	1.046 (0.577–1.895)	0.882
	College and above	38 (17.4)	35 (17.9)	0.677 (0.378–1.213)	0.190^∗^

Occupation	Civil servant	47 (21.6)	43 (22.1)	1	
	Farmer	55 (25.2)	62 (31.7)	2.287 (0.668–7.834)	0.188^∗^
	Merchant	37 (17.0)	30 (15.4)	2.818 (0.836–9.498)	0.095^∗^
	Housewife	27 (12.4)	21 (10.8)	2.027 (0.578–7.114)	0.270
	Student	42 (19.3)	35 (17.9)	1.944 (0.534–7.079)	0.313
	Other	10 (4.6)	4 (2.1)	2.083 (0.601–7.223)	0.247^∗^

Contact lens wearing	Yes	21 (9.6)	13 (6.7)	1.492 (0.726–3.067)	0.276
	No	197 (90.4)	182 (93.3)	1	

History of eye surface disease	Yes	114 (52.3)	89 (45.6)	0.766 (0.520–1.128)	0.177^∗^
	No	104 (47.7)	106 (54.4)	1	

Hospital admission ever?	Yes	112 (51.4)	97 (49.7)	1.067 (0.725–1.571)	0.740
	No	106 (48.6)	98 (50.3)	1	

History of eye trauma	Yes	72 (33.0)	58 (29.7)	1.165 (0.768–1.768)	0.473
	No	146 (67.0)	137 (70.3)	1	

Previous use of antibiotics	Yes	130 (59.6)	90 (46.2)	0.580 (0.393–0.857)	0.006^∗^
	No	88 (40.4)	105 (53.8)	1	

Duration of illness (*n* = 220)	< 1 week	32 (17.3)	12 (34.3)	1	
	2–4 weeks	66 (35.7)	12 (34.3)	2.966 (1.190–7.390)	0.020^∗^
	> 4 weeks	87 (47.0)	11 (31.4)	1.438 (0.597–3.462)	0.418

Comorbid condition^∗∗^	Yes	56 (25.7)	34 (17.4)	0.611 (0.379–0.986)	0.044^∗^
	No	162 (74.3)	161 (82.6)	1	

Use of a digital screen	Yes	37 (17.0)	30 (15.4)	1.124 (0.665–1.902)	0.662
	No	181 (83.0)	165 (84.6)	1	

Eye cosmetics usage	Yes	21 (9.6%)	15 (7.7)	1.279 (0.640–2.557)	0.486
	No	197 (90.4)	180 (92.3)	1	

Abbreviations: % = percentage, CI = confidence interval, COR = crude odds ratio, N = number.

^∗^Statistically significant.

^∗∗^Comorbid conditions: systemic diseases like diabetes mellitus, hypertension, renal diseases, and heart diseases.

**Table 5:** Multivariable logistic regressions of sociodemographic, clinical, and behavioral factors assessed against culture-confirmed external eye infections at DUGH ophthalmic clinic, Southern Ethiopia, 2023.

Variables	Categories	Bacterial isolates *N* (%)		AOR (95% CI)	*p*-value
		Yes	No		
Age	≤ 14	49 (22.5)	60 (30.8)	1	
	15–24	20 (9.2)	12 (6.2)	7.016 (0.954–51.579)	0.056
	25–64	130 (59.6)	102 (52.3)	1.475 (0.175–12.396)	0.721
	≥ 65	19 (8.7)	21 (10.8)	1.187 (0.210–6.708)	0.846

Sex	Female	119 (54.6)	103 (52.8)	—	—
	Male	99 (45.4)	92 (47.2)		

Residence	Urban	100 (45.9)	81 (41.5)	—	—
	Rural	118 (54.1)	114 (58.5)		

Educational level	Illiterate	48 (22.1)	59 (30.3)	1	
	Primary	55 (25.2)	53 (27.2)	1.827 (0.206–16.202)	0.588
	Secondary	77 (35.3)	48 (24.6)	0.863 (0.091–8.179)	0.898
	College and above	38 (17.4)	35 (17.9)	0.470 (0.091–2.429)	0.368

Occupation	Civil servant	47 (21.6)	43 (22.1)	1	
	Farmer	55 (25.2)	62 (31.7)	1.199 (0.093–15.441)	0.889
	Merchant	37 (17.0)	30 (15.4)	4.277 (0.381–47.986)	0.239
	Housewife	27 (12.4)	21 (10.8)	1.930 (0.165–22.512)	0.600
	Student	42 (19.3)	35 (17.9)	1.761 (0.138–22.535)	0.664
	Other	10 (4.6)	4 (2.1)	0.328 (0.019–5.674)	0.443

Contact lens wearing	Yes	21 (9.6)	13 (6.7)	—	—
	No	197 (90.4)	182 (93.3)		

History of eye surface disease	Yes	114 (52.3)	89 (45.6)	11.794 (2.799–49.692)	0.001^∗^
	No	104 (47.7)	106 (54.4)	1	

Hospital admission ever?	Yes	112 (51.4)	97 (49.7)	—	—
	No	106 (48.6)	98 (50.3)		

History of eye trauma	Yes	72 (33.0)	58 (29.7)	—	—
	No	146 (67.0)	137 (70.3)		

Previous use of antibiotics	Yes	130 (59.6)	90 (46.2)	3.471 (1.123–10.725)	0.031^∗^
	No	88 (40.4)	105 (53.8)	1	

Duration of illness (*n* = 220)	< 1 week	32 (17.3)	12 (34.3)	1	
	2–4 weeks	66 (35.7)	12 (34.3)	1.249 (0.324–4.823)	0.747
	> 4 weeks	87 (47.0)	11 (31.4)	0.407 (0.115–1.442)	0.164

Comorbid condition	Yes	56 (25.7)	34 (17.4)	2.066 (0.615–6.944)	0.241
	No	162 (74.3)	161 (82.6)	1	

Use of a digital screen	Yes	37 (17.0)	30 (15.4)	—	—
	No	181 (83.0)	165 (84.6)		

Eye cosmetics usage	Yes	21 (9.6%)	15 (7.7)	—	—
	No	197 (90.4)	180 (92.3)		

Abbreviations: % = percentage, AOR = adjusted odds ratio, CI = confidence interval, N = Number.

^∗^Statistically significant.

^∗∗^Comorbid conditions: systemic diseases like diabetes mellitus, hypertension, renal diseases, and heart diseases.

## Data Availability

Data will be made available from the corresponding author on request.

## Nomenclature

AOR: Adjusted odds ratio
ATCC: American Type Culture Collection
CLSI: Clinical Laboratory Standard Institute
BHI: Brain heart infusion
CONS: Coagulase-negative staphylococci
DUGH: Dilla University General Hospital
EEI: External eye infection
EPHI: Ethiopian Public Health Institute
MDR: Multidrug resistance
MRSA: Methicillin-resistant Staphylococcus aureus
SPSS: Statistical Package for Social Sciences.
